# An open chat with…Takashi Gojobori

**DOI:** 10.1002/2211-5463.13354

**Published:** 2022-01-17

**Authors:** Duncan Wright, Takashi Gojobori

**Affiliations:** ^1^ FEBS Open Bio Editorial Office Cambridge UK; ^2^ Computer, Electrical and Mathematical Sciences and Engineering Division (CEMSE) Computational Bioscience Research Center (CBRC) King Abdullah University of Science and Technology (KAUST) Thuwal Saudi Arabia

## Abstract

Takashi Gojobori was one of the original founding members of the Editorial Board of *FEBS Open Bio* and is easily one of our most hard‐working editors, having carefully evaluated hundreds of manuscripts on bioinformatics for us over the last decade. Takashi Gojobori received his doctorate at Kyushu University, Japan, before joining the University of Texas Health Science Center as a Research Associate and then Research Assistant Professor. Takashi was formerly the Vice‐Director and Professor of National Institute of Genetics (NIG), Mishima, Japan, and at present, he is Distinguished Professor of Bioscience and Bioscience Acting Director of the Computational Bioscience Research Center at King Abdullah University of Science and Technology (KAUST), Saudi Arabia. Takashi was formerly the Editor‐in‐Chief of the journal *Gene* and is also a long‐term member of the Editorial Board of *FEBS Letters*, among other editorial appointments. In honour of his 10 years (and counting!) of service to the journal, we present here this interview with him on his research and experiences.

## Graphical abstract

Takashi Gojobori was one of the original founding members of the Editorial Board of *FEBS Open Bio* and is easily one of our most hard‐working editors, having carefully evaluated hundreds of manuscripts on bioinformatics for us over the last decade. In honour of his 10 years (and counting!) of service to the journal, we present here this interview with him on his research and experiences.

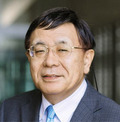

## For those who are nonspecialists, how would you describe your research?

I am working on the research of comparative and evolutionary genomics by comparing genomic and genomic‐related information among species. I try to elucidate the evolutionary origin and processes of particular genomic regions and genes of interest. By doing so, I try to deepen the understanding of molecular mechanisms and the network of biological components in various organisms.

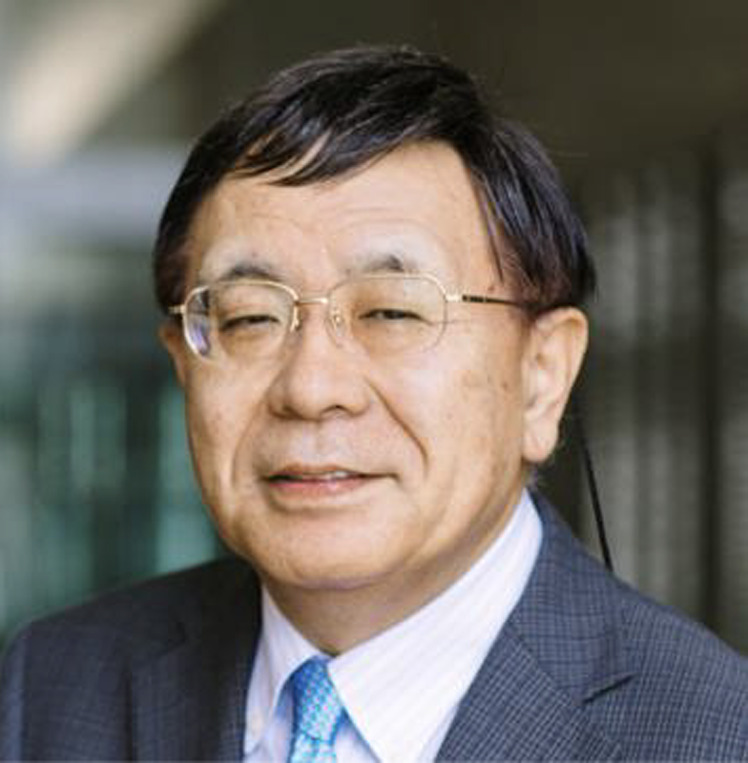

## Of all the papers you've authored in your career, which are your favourites?

One of my favourite publications is the PNAS paper (1985) in which I reported the discovery that the rate of nucleotide substitution for retroviruses such as HIV is a million times higher than that of humans and other mammals [1].

Additionally, I published a *Nature Genetics* paper (2004) that reported the discovery that about 14% of bacterial genomes originated from different species through horizontal gene transfer [2].

These discoveries led to the recognition of evolutionary features of genomes that are very flexible and generous in evolutionary terms.

## You were the doctoral supervisor of Emperor Naruhito's younger brother, Prince Akishino. Was supervising such a high‐profile student a daunting task?

It was almost 25 years ago that I came to know Emperor Naruhito's younger brother, Prince Akishino. Because he was such an excellent and diligent student, it was not so difficult for him to obtain a Ph.D. under my supervision. Many security guards were always surrounding him whenever he visited my laboratory, and it was quite fun to have such additional extraordinary experiences.

## I understand you also worked at the Tokyo Imperial Palace and co‐authored publications with the former Emperor of Japan, Emperor Akihito. How did you find working at the Palace with the Emperor?

I collaborated with the former Emperor of Japan, Emperor Akihito, for almost 25 years when we worked on writing research papers; therefore, I have regularly been invited to the Palace. He is very knowledgeable about various subjects in biology in general. In particular, he is one of the world's leading fish taxonomists, and I learned a lot from him. Some meetings with Emperor Akihito lasted 2–3 h. We would have a break in the middle, and then, Empress Michiko would bring tea and cakes to us by herself. It was great fun for her to support his research in such a thoughtful manner.

## What do you consider to be the most exciting recent developments in bioinformatics?

The most exciting recent development in bioinformatics is the active utilization of Artificial Intelligence (AI). It will be interesting to address the difficult questions in bioinformatics by incorporating AI.

## What advice would you give young people interested in a research career today?

I would like to advise young people to challenge the difficult but significant problems without any hesitation. I would like all young people to keep in mind that our questions are more important than answered ones.

## What sparked your interest in phylogenetics and the at the time nascent field of bioinformatics?

When I started my research as a Ph.D. student and postdoc fellow, I liked the so‐called mathematical biology. Although there was no term for ‘bioinformatics’, at the time, I wanted to use computers for conducting data analyses in biology. I was attracted by theoretical population genetics and molecular evolution because I had to use a computer for the data analysis of evolutionary studies of gene and genomics sequenced data. It naturally led me to phylogenetics, which became one of the fields of bioinformatics later.

## You are currently affiliated with King Abdullah University of Science and Technology (KAUST) in Saudi Arabia. How does the research culture at KAUST compare with that of the National Institute of Genetics in Japan?

I have been affiliated with King Abdullah University of Science and Technology (KAUST) in Saudi Arabia since 2013.

I like this university because the faculty originate from different parts of the world, and their research interests are pretty diverse. Because of this heterogeneity, I am always inspired when I have discussions with colleagues. I think that KAUST has such a great advantage over other institutes as we work on a very unique and significant agenda of science.
